# Correlation between hypothalamic-pituitary-adrenal function and clinical characteristics in the acute phase of first-episode drug-naive major depressive disorder

**DOI:** 10.3389/fpsyt.2026.1985217

**Published:** 2026-10-26

**Authors:** Liangsheng Liu, Xing Yang, Duoduo Lin

**Affiliations:** 1 Fujian Medical University, Graduate School, Fuzhou, Fujian, China; 2 Xiamen Xianyue Hospital, Xianyue Hospital Affiliated with Xiamen Medical College, Fujian Psychiatic Center, Fujian Clinical Research Center for Mental Disorder, Xiamen, Fujian, China

**Keywords:** HAMD-24, hypothalamic-pituitary-adrenal axis, major depressive disorder, SDS, serum cortisol

## Abstract

**Background:**

First-episode drug-naïve depressive disorders have high heterogeneity in clinical features, and there is still room for in-depth exploration of the correlation with serum cortisol levels. This study aimed to explore the correlation between hypothalamic-pituitary-adrenal (HPA) function in the acute phase of first-episode drug-naïve major depressive disorder (MDD) and clinical features evaluated by the Hamilton Depression Rating Scale (HAMD-24) and the Zung Self-Rating Depression Scale (SDS).

**Methods:**

Patients with first-episode drug-naïve MDD in the acute phase meeting the DSM-V diagnostic criteria were selected as the study group, and healthy individuals matched for gender and age were recruited as the control group. Serum cortisol levels were measured in both groups, and patients in the MDD group were evaluated using HAMD-24 and SDS.

**Results:**

The serum cortisol level in the MDD group(307.03 ± 77.80 nmol/L) was significantly higher than that in the healthy control group(261.13 ± 58.15 nmol/L) (Z-Value = -3.539, P < 0.05). The serum cortisol level showed a positive correlation trend (ρ > 0) with the anxiety factor, cognitive impairment factor, retardation factor, despair factor, total HAMD score, and SDS score, with statistical significance (p < 0.05). It showed a negative correlation trend (ρ < 0) with the sleep factor, without statistical significance (p > 0.05). It also showed a positive correlation trend (ρ > 0) with the weight factor and diurnal variation factor, without statistical significance (p > 0.05).

**Conclusion:**

A significant increase in serum cortisol level can serve as one of the biological indicators for predicting the core symptom clusters and disease severity of MDD. Regulation of The HPA axis cortisol pathway may have potential value in alleviating MDD symptoms.

## 1 Introduction

Major Depressive Disorder (MDD) is a type of psychiatric and psychological disorder characterized by significant and persistent low mood caused by various factors. Its core symptoms include low mood, anhedonia, and decreased energy, accompanied by symptoms such as anxiety, low self - evaluation, pessimism, sleep disorders, and appetite changes (1). The global point prevalence is approximately 5%, and the lifetime prevalence ranges from 10% to 15% (2). MDD is an emotional disorder with high recurrence rate, high disability rate, and high suicide risk (3), imposing a huge global public health burden. By 2021, among the 12 subtypes of mental disorders worldwide, major depressive disorder (MDD) had the highest age - standardized disability - adjusted life years (DALYs), accounting for 30.01% of the total DALYs of global mental disorders (4). It is one of the main diseases causing functional disability in humans.

The Hypothalamic-Pituitary-Adrenal (HPA) axis is the core neuroendocrine pathway of the body’s stress response, and its dysfunction has been widely recognized as the key biological basis for the onset and recurrence of depressive disorders (5, 6). In the past five years, research has expanded the close relationship between the HPA axis and depression on the classic framework of “excessive activation causes impaired negative feedback, which in turn leads to neurotoxicity”, and derived new rapid antidepressant targets targeting glucocorticoid receptors (GR) and corticotropin-releasing hormone (CRH) receptors (7). Changes in serum cortisol levels are an intuitive manifestation of the terminal response of HPA functional changes. A large amount of clinical evidence shows that approximately 60% - 70% of patients with major depressive disorder (MDD) exhibit elevated serum cortisol and disrupted nocturnal rhythms (8, 9), which are considered one of the key biomarkers of MDD.

Currently, the objective assessment of the severity of major depressive disorder (MDD) can be carried out through scale scoring. The Hamilton Rating Scale for Depression (HAMD) (10, 11) is one of the main scales currently used in clinical practice. The HAMD-24 has 24 items, providing a more comprehensive assessment. According to the item categories, it consists of seven factors: anxiety/somatization, weight, cognitive impairment, diurnal variation, retardation, sleep disorder, and hopelessness. Each factor reflects different clinical features of depression, and different clinical features indicate different clinical intervention measures and prognoses. Timely and reasonable clinical intervention may have a positive impact on the prognosis, making reasonable assessment particularly important. The Zung Self-Rating Depression Scale (SDS) (12, 13), as one of the self-rating scales for depressive symptoms, can reflect the subjective severity of depressive symptoms and is also of great significance for the assessment of MDD. At present, studies on the correlation between changes in hypothalamic-pituitary-adrenal (HPA) axis function, which leads to changes in serum cortisol levels, and HAMD-24 and SDS mainly focus on the total scores. There are still relatively few specific studies on the correlation between each factor of HAMD-24 and serum cortisol levels. MDD is highly heterogeneous (14, 15), which may be related to different clinical and molecular characteristics caused by different neuroanatomical subtypes (16). Existing studies have not fully, objectively, and truly reflected the clinical characteristics of MDD, leaving room for further research.

This study focuses on further exploring the correlations between serum cortisol levels and each factor and the total score of HAMD - 24, as well as SDS in patients with first - episode, drug - naive major depressive disorder (MDD) in the acute episode phase. The aim is to clarify the interrelationships more precisely. That is, from the perspective of the biological indicator of serum cortisol, we aim to predict the clinical characteristics and severity of MDD, providing more reference for clinical practice. This can remind medical staff to provide timely clinical interventions, reduce negative risks such as self - injury, self - mutilation, and suicide, and relieve the pain of MDD patients caused by various clinical symptoms, which has positive and significant implications. The method of this study refers to the core points method proposed by experts such as Soares JC (17) for building a learning - based health network related to mood disorders including MDD, which includes the severity of symptoms, objective functional assessment data such as neuropsychological tests, and blood markers.

## 2 Materials and methods

### 2.1 Participants

Sample size was calculated *a priori* using GPower 3.1.9.7 (18). We set a two-tailed α of 0.05 and statistical power (1−β) of 0.85. The hypothesized population correlation coefficient (ρ = 0.30) was derived from a previous clinical study in first-episode drug-naïve patients with major depressive disorder (19). The minimal analyzable sample size calculated was 93, we recruited 103 participants.

A total of 103 patients aged 18–60 years old with first - episode major depressive disorder in the acute phase were recruited from the outpatient department of our hospital as the study subjects, including 42 males and 61 females. The control group consisted of 99 healthy individuals who had physical examinations in our hospital during the same period, including 39 males and 60 females.

### 2.2 Enrollment criteria

MDD group: a. Meeting the diagnostic criteria for the first-episode acute phase of major depressive disorder in the Diagnostic and Statistical Manual of Mental Disorders (5th Edition), namely DSM-V; b. Having never used any form of antidepressant drugs or drugs with antidepressant effects; c. Having never received any form of psychotherapy or physical therapy such as modified electroconvulsive therapy without convulsions (MECT) and repetitive transcranial magnetic stimulation therapy (rTMS); d. Having no clinically significant somatic diseases such as cardiovascular and cerebrovascular system diseases, neuroendocrine system diseases, and hematological system diseases; e. Having no abuse of any kind of psychoactive substances, hormonal drugs, etc.; f. Patients having no family history of any form of mental illness.

Healthy control group: All participants have no somatic or psychological diseases, and there are no clinically significant abnormalities in neuropsychological tests.

### 2.3 Determination of serum cortisol levels

Both subjects in the depression group and the control group had 10 ml of venous blood drawn at 8 a.m. on an empty stomach in the early morning for the determination of serum cortisol levels. Measurement method: Serum cortisol was measured by competitive-binding electrochemiluminescence immunoassay on Roche cobas e 402/e 801 analyzers with manufacturer-supplied reagents. The assay was traceable to ID-GC-MS reference method, with a measuring range of 1.5-1750 nmol/L and acceptable within-run and intermediate precision. Specimens were assayed within 2 hours of venipuncture; lipemic or hemolytic samples were discarded. Quality-control procedures were strictly implemented throughout the study. The assessment of psychological scales is conducted by full-time assessment physicians from the Psychological Assessment Center of our hospital who have received professional training.

### 2.4 Ethical approval

The study was approved by the Medical Ethics Committee of our hospital (Approval No.: 2024-KY-006), and all study subjects signed relevant informed consent forms.

## 3 Statistical test

The SPSS 26 statistical software was used to analyze the data. The Shapiro - Wilk test was employed to determine whether the data followed a normal distribution. A p - value ≥ 0.05 indicated a normal distribution, while a p - value < 0.05 indicated a non - normal distribution. For inter - group comparison, parametric tests (t - test, ANOVA) were used for data conforming to the normal distribution, and non - parametric tests (Mann-Whitney U test) were performed for non - normally distributed data. For correlation analysis between continuous variables, Pearson r analysis was used for data following a normal distribution, and Spearman ρ analysis was used for non - normally distributed data. Based on the scatter plots of continuous variables, for data showing a non - linear trend distribution, stratified multiple quadratic polynomial regression was further used to analyze whether there was a non - linear curve association between variables. Before further analysis, the original serum cortisol was mean - centered to obtain Cort_c, and then the centered cortisol quadratic term Cort_c2 was constructed to reduce the multicollinearity between the first - order and second - order terms. Three nested models were constructed in a stratified manner: Model 1 included covariates (age, gender, disease duration); Model 2 added the first - order term of centered cortisol Cort_c on the basis of Model 1 to test the linear association; Model 3 further introduced the centered quadratic term Cort_c2 to test the existence of a quadratic curve association. The model fitting was evaluated by R2, Adjusted R2, △R2, and Fchange. All tests were two - sided, and P < 0.05 was considered statistically significant, the P values were adjusted for multiple comparisons using the Benjamini–Hochberg false discovery rate (FDR) method. Adjusted P values are presented (https://www.sdmproject.com/utilities/?show=FDR).

## 4 Results

### 4.1 Demographic and clinical characteristics

There was no significant difference in age between the depression group (28.01 ± 9.01 years) and the control group (28.89 ± 7.69 years) (Z = 1.687, p > 0.05). After the chi-square test, there was no significant difference in gender between the two groups (χ^2^ = 0.04, P > 0.05). The HAMD-24 score (34.06 ± 6.29) and SDS score (71.35 ± 8.89) of the MDD group both reached the diagnostic scores. The average course of MDD was 4.36 ± 2.51 months (**Table 1**, presented later in this article).

**Table 1:** Comparison of gender, age, serum cortisol between MDD and control.

					*Metrics*			
Variables					*χ^2^*	*df*	*Z-value*	*P-value*
Group	MDD		Control					
Gender	male (n)	female (n)	male (n)	female (n)				
	42	61	39	60	0.04	1		0.886

	mean ± SD							
Age	28.01 ± 9.01		28.89 ± 7.69				1.687	0.092
Serum Cortisol	307.03 ± 77.80^*^		261.13 ± 58.15				-3.539	0.000
Mean Duration	4.36 ± 2.51							
HAMD	34.06 ± 6.29							
SDS	71.35 ± 8.89							

*indicates statistically significant compared with the control group, P<0.05 is statistically significant, gender used χ^2^ test, age and serum cortisol used Mann-Whitney U test; age unit is year, serum cortisol unit is nmol/L, duration unit is month.

### 4.2 Comparison of serum cortisol levels between the depression group and the healthy control group

The results showed that the serum cortisol level in patients with major depressive disorder (MDD) (307.03 ± 77.80 nmol/L) was significantly higher than that in the control group (261.13 ± 58.15 nmol/L) (p < 0.05) (**Table 1**).

### 4.3 Correlation analysis between serum cortisol levels and HAMD - 24 factors and total score in MDD

In patients with first-episode, drug-naïve major depressive disorder in the acute phase, serum cortisol levels were significantly correlated with the HAMD-24 anxiety factor (ρ=0.642, P<0.05), cognitive impairment factor (ρ=0.365, P<0.05), retardation factor (ρ=0.481, P<0.05), despair factor (ρ=0.399, P<0.05), and total HAMD score (ρ=0.602, P<0.05),the above FDR-adjusted P values were <0.05. There was a tendency toward a positive correlation with the weight factor (ρ=0.082, P>0.05) and the diurnal variation factor (ρ=0.133, P>0.05), but the correlations were not significant. There was a tendency toward a negative correlation with the sleep factor (ρ=-0.087, P>0.05), these three FDR-adjusted P values were>0.05,and the correlation was also not significant (**Figures 1A–H**, presented later in this article; **Table 2**).

**Figure 1:**
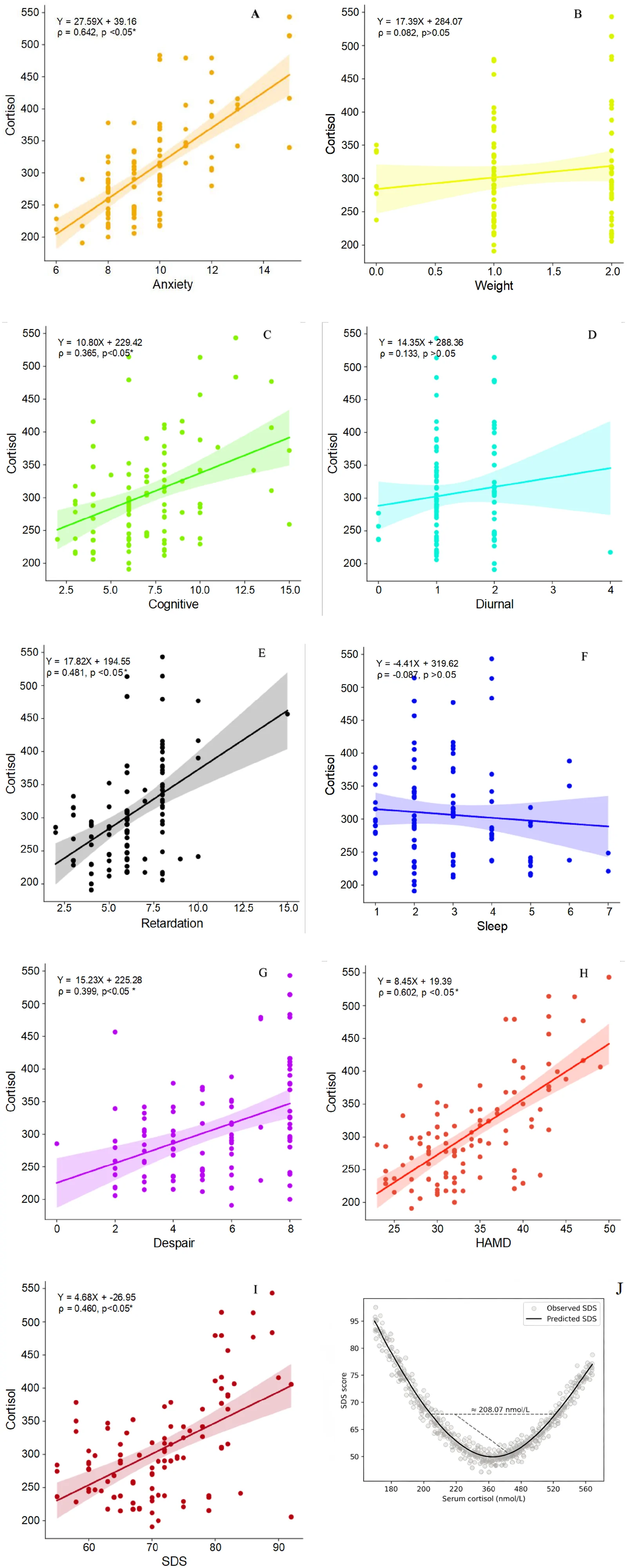
**(A–I)** Scatter plot of correlation analysis between serum cortisol and various factors of HAMD-24 and SDS; The J-graph shows a U-shaped curve correlation between serum cortisol levels and SDS scores after adding covariates: age, gender, and disease duration.

**Table 2:** Correlations of serum cortisol with HAMD-24 subfactors, total HAMD and SDS score.

Statistical parameters	ρ	BCa (95% CI)	*P*	FDR-adjusted *P*
Anxiety	0.642	(0.501,0.750)	0.000	0.000
Weight	0.082	(-0.108,0.266)	0.410	0.410
Cognitive	0.365	(0.177,0.534)	0.000	0.000
Diurnal	0.133	(-0.069,0.331)	0.179	0.230
Retardation	0.481	(0.278,0.635)	0.000	0.000
Sleep	-0.087	(-0.272,0.112)	0.383	0.410
Despair	0.399	(0.210,0.568)	0.000	0.000
HAMD	0.602	(0.429,0.737)	0.000	0.000
SDS	0.460	(0.251,0.634)	0.000	0.000

ρ denotes the Spearman correlation coefficient. BCa (95% CI) is the abbreviation for Bias-Corrected and Accelerated bootstrap confidence interval. FDR refers to the False Discovery Rate adjusted *P*-value. Statistical significance was defined at FDR-adjusted *P* < 0.05.

### 4.4 Correlation analysis between serum cortisol levels and SDS score

In drug - naive patients with first - episode major depressive disorder (MDD), the serum cortisol level was positively correlated with the Self - Rating Depression Scale (SDS) score (ρ = 0.460, P < 0.05,FDR-adjusted P< 0.05) (**Table 2**).

### 4.5 Hierarchical multiple quadratic polynomial regression analysis

After further analysis using stratified multiple quadratic polynomial regression and correction for age, gender, and disease duration, in Model 2, the P_F-change_ values between serum cortisol levels and the anxiety factor, cognitive impairment factor, retardation factor, despair factor, and total HAMD score were all <0.05. However, in Model 1 and Model 3, the P_F-change_ values were >0.05, indicating a linear correlation with these five factors. No significant non - linear association in the form of a quadratic curve was detected. The above analysis results suggest that as the serum cortisol level increases, symptoms related to anxiety, cognitive impairment, retardation, despair, and overall depressive symptoms tend to worsen. There is no evidence to suggest that the symptoms will change in the opposite direction after cortisol reaches a certain inflection point. Regarding the correlation with SDS, in Model 1, the P_F-change_ value was >0.05, while in Model 2 and Model 3, the P_F-change_ values were <0.05, indicating a significant non - linear association in the form of a quadratic curve. Based on the regression coefficients, this curve was judged to be U - shaped. Calculated according to the unstandardized regression coefficients in the stratified quadratic polynomial regression Model 3 with covariates corrected: B_cortisol_c_=0.038,B_cortisol_c2_ ≈ 0.00019,substitute into the inflection point calculation formula: $X_{cent\_\inf lection}=-\frac{B_{cortisol\_c}}{2\times B_{cortisol\_c2}}$, The inflection point corresponding to the U-shaped curve has a centered cortisol value of -98.96, which is equivalent to an original serum cortisol level of 208.07 nmol/L. When the cortisol level is below this inflection point, the SDS score decreases as the serum cortisol level increases; when it exceeds the inflection point, the SDS score increases as the cortisol level rises. This inflection point is obtained from the fitting within the sample of this study and is not suitable to be directly used as a clinical cut-off value (**Table 3**, **Supplementary Table 1**, **Figure 1J**, presented later in this article).

**Table 3:** Model summary for hierarchical second-order polynomial regression across all outcome measures.

Outcome measure	Model	Variables entered	R2	Adjusted R2	△R2	F*_change_*	P*_F-change_*
Anxiety	Model 1	A	0.047	0.018	0.047	1.635	0.186
	Model 2	B	0.487	0.467	0.44	84.193	0.000*
	Model 3	C	0.489	0.463	0.002	0.367	0.546
Weight	Model 1	A	0.034	0.005	0.034	1.155	0.331
	Model 2	B	0.043	0.004	0.010	0.981	0.324
	Model 3	C	0.077	0.029	0.033	3.493	0.065
Cognitive	Model 1	A	0.012	-0.018	0.012	0.406	0.749
	Model 2	B	0.177	0.143	0.165	19.603	0.000*
	Model 3	C	0.178	0.136	0.002	0.179	0.673
Diurnal	Model 1	A	0.054	0.025	0.054	1.883	0.137
	Model 2	B	0.062	0.024	0.008	0.856	0.357
	Model 3	C	0.069	0.021	0.007	0.707	0.402
Retardation	Model 1	A	0.020	-0.010	0.020	0.657	0.580
	Model 2	B	0.244	0.213	0.225	29.127	0.000*
	Model 3	C	0.244	0.205	0.000	0.006	0.937
Sleep	Model 1	A	0.022	-0.007	0.022	0.785	0.520
	Model 2	B	0.036	-0.004	0.013	1.359	0.246
	Model 3	C	0.061	0.012	0.025	2.563	0.113
Despair	Model 1	A	0.020	-0.010	0.020	0.669	0.573
	Model 2	B	0.175	0.141	0.155	18.442	0.000*
	Model 3	C	0.180	0.137	0.004	0.526	0.470
HAMD Score	Model 1	A	0.025	-0.005	0.025	0.836	0.477
	Model 2	B	0.467	0.445	0.442	81.166	0.000*
	Model 3	C	0.473	0.445	0.006	1.119	0.293
SDS	Model 1	A	0.072	0.044	0.072	2.568	0.059
	Model 2	B	0.313	0.284	0.240	34.256	0.000*
	Model 3	C	0.359	0.326	0.047	7.064	0.009*

A represents covariates including age, gender and duration, B represents A+Cort_c, C represents A+Cort_c2.

## 5 Discussion

Research suggests that the abnormal function of the hypothalamic-pituitary-adrenal (HPA) axis is one of the etiologies and pathogenesis mechanisms of major depressive disorder (MDD). During the acute episode of depression, the levels of serum cortisol, the terminal effector of the HPA axis, significantly increase, accompanied by disruptions in the circadian secretion rhythm (20–22). Our previous study also found a significant increase in serum cortisol levels during the acute episode of depression (23). When exposed to stress, the function of the HPA axis is activated. If the intensity or duration of the stress exceeds the normal response capacity of the body, the HPA axis may become hyperactivated, show rhythm disturbances, or even enter a state of exhaustion after over - activation (24, 25).A large amount of evidence - based medical evidence shows that before the onset of many cases of MDD, patients often experience various stressful events. Even before the appearance of obvious depressive symptoms, the HPA axis is over - activated, leading to an increase in serum cortisol levels (26). Through the feedback mechanism, elevated cortisol not only affects the HPA axis itself but may also further influence the changes in neurotransmitters related to the onset of MDD (27, 28), which in turn affects the controllability of brain function and promotes the emergence of various clinical features of MDD (29). Moreover, a depressive episode itself is a significant negative stress event, which may exacerbate the dysregulation of the HPA axis function. This reciprocal “damage” poses challenges to the treatment and recovery of MDD. This might be one of the reasons why the response rate of existing antidepressants with known mechanisms is only about 48%–50%, and the specific efficacy is only about 18%–20% (30). It also partially explains why many MDD patients in clinical practice are unable to fully recover to their pre - illness states.

In this study, we found that in patients with first-episode, drug-naïve major depressive disorder (MDD) in the acute phase, serum cortisol was significantly positively correlated with the anxiety factor of the 24-item Hamilton Depression Rating Scale (HAMD-24). The anxiety factor consists of psychic anxiety, somatic anxiety, gastrointestinal symptoms, general symptoms, hypochondriasis, and insight, covering both mental and physical symptoms. It is more suitable for understanding the intrinsic relationship between the hypothalamic-pituitary-adrenal (HPA) axis and the “anxious depression” phenotype (19, 31). From the perspective of pathophysiological mechanisms, the HPA axis is the core pathway for the body to cope with acute stress. Acute stress prompts the hypothalamus to release corticotropin-releasing hormone (CRH), which in turn stimulates the secretion of adrenocorticotropic hormone (ACTH) and cortisol. When the negative feedback regulation function of cortisol is impaired, the peripheral cortisol level continues to rise, which can amplify the over - arousal of the amygdala in the limbic system, inducing psychic anxiety. At the same time, it mediates somatic manifestations such as gastrointestinal discomfort, general aches, and hypochondriasis through the autonomic nervous system, corresponding to the multi-dimensional symptoms included in the HAMD - 24 anxiety factor (32, 33). Previous studies on depression with somatic symptoms have confirmed that the plasma cortisol level of depressive patients with significant somatic symptoms is significantly higher than that of those without somatic symptoms, and the increase in cortisol is closely related to the somatic - related symptoms of HAMD - 24, supporting the specific role of cortisol in the somatic anxiety symptoms of depression. The clinical significance of the results of this study lies in revealing that the increase in serum cortisol can serve as a potential peripheral biomarker for identifying the anxiety subtype of first-episode depression. In MDD patients with prominent somatization symptoms of anxiety, the hyperactivity of the HPA axis may be more significant. There are differences in the response characteristics of this population to different antidepressant drugs and psychological interventions, and clinical interventions should be individualized.

Meanwhile, it was also found that in this type of MDD patients, the serum cortisol level was significantly positively correlated with the scores of the cognitive impairment factor. The cognitive impairment factor includes six items: guilt, suicide, agitation, depersonalization/derealization, paranoid symptoms, and obsessive - compulsive symptoms, suggesting that the hyperactivity of the HPA axis is closely related to cognitive distortion and psychotic - like symptoms in the acute phase of MDD. The possible pathological mechanism is that continuously elevated cortisol acts on the brain regions involved in emotion and cognitive regulation and causes the dysregulation of the negative feedback mechanism, leading to the up-regulation of the activity of the mesencephalic dopaminergic pathway, mediating damage to neural brain regions such as the hippocampus (34, 35), impairing cognitive control and reality-testing ability, and inducing the occurrence of the above symptoms (36–38). This study avoided the interference of various confounding factors and can reflect the association between serum cortisol levels and cognitive symptoms in the early stage of the disease. Similar previous studies have also found that elevated cortisol is significantly correlated with the HAMD cognitive-related sub - scores including items such as suicide and paranoia, indicating that HPA axis abnormalities are early biological markers of high-risk cognitive symptoms (39). Preclinical and clinical studies have proposed that cortisol dysregulation may disrupt prefrontal-limbic circuit information processing via impaired brain glymphatic function, which could potentially reinforce maladaptive cognitive patterns including self-blame, paranoia and rumination, and thereby contribute to functional impairment (40–42). Of note, this mechanistic pathway remains speculative and is not directly validated by the present data. The clinical significance is that serum cortisol can assist in identifying the high - risk subgroup with cognitive distortion, suicidal ideation, and paranoid - dissociative symptoms in first - episode MDD. Patients with prominent cognitive abnormalities have a higher suicide risk, and the response characteristics of conventional antidepressants may be different from those of other subtypes. Intervention strategies targeting the HPA axis (such as glucocorticoid receptor modulators) may become the individualized treatment direction for this subgroup.

In this study, the serum cortisol level was also significantly positively correlated with the retardation factor. The retardation factor consists of depressive mood, decreased work and interest, psychomotor retardation, and sexual symptoms, and contains the core symptom cluster of MDD, suggesting that the increase in serum cortisol plays an important role in exploring the high risk of typical depressive episodes. Some studies have also shown that depressive mood, as an important component of the retardation factor, is an important indicator for judging melancholic depression (43). This association between neuroendocrine and clinical symptoms provides a biological basis for understanding the heterogeneity of MDD and is also consistent with the MDD subtypes retained in the current DSM-V (44).

Similarly positively correlated is the relationship between serum cortisol levels and the despair factor. This factor is composed of a sense of diminished ability, despair, and inferiority, and it comprehensively reflects the core negative self - cognition and despair emotions in depression. The possible reason is that high levels of serum cortisol can damage the neural plasticity of the hippocampus and prefrontal cortex. These brain regions are strongly associated with the clinical symptoms of major depressive disorder (MDD), leading individuals to exhibit typical depressive manifestations such as reduced self-evaluation, loss of ability, and pessimistic hopelessness (24, 45–47). Previous studies have also confirmed that abnormally elevated cortisol levels in patients with first-episode MDD are closely related to hopeless cognition, low self - esteem, and pessimistic emotions, which can exacerbate the core symptoms of depression and increase the risk of poor prognosis (48, 49). This result further refines the regulatory characteristics of cortisol on the heterogeneous symptoms of MDD. Clinically, patients with first-episode MDD who have high cortisol levels accompanied by significant despair symptoms may have a worse prognosis and a higher risk of suicide, providing a reference for the accurate identification of depressive subtypes and individualized interventions.

Regarding the overall symptoms of MDD, our study also found a significant positive correlation between serum cortisol levels and the total score of HAMD - 24. HAMD - 24 is an observer- rated scale, and its total score tends to reflect the objective severity of MDD. In contrast, SDS is a self - rated scale, which reflects the severity of patients’ subjective depressive mood and is closely related to the subjective pain experience. The changing trend of serum cortisol is consistent with both, indicating its consistency between the subjective experience and objective manifestation in MDD patients, suggesting that the degree of HPA axis hyperactivity is closely related to the overall severity of depression. Further analyses were performed within the present study sample revealed a U - shaped correlation between serum cortisol levels and SDS, showing a concentration-dependent bidirectional effect (50). This reflects that a moderate level of cortisol can maintain the normal stress - buffering and emotion - regulation functions of the body. Either too low or too high cortisol levels may affect the body’s compensatory ability and damage the brain regions involved in emotion regulation, both of which can exacerbate the subjective pain of MDD. This is a novel finding in our study. The individual differences in glucocorticoid receptor sensitivity can explain this phenomenon: Some first - episode depressive patients may develop glucocorticoid resistance, while others show a blunted HPA axis response, both of which can exacerbate the clinical manifestations of depression (51–53). Studies on untreated MDD also confirmed the association between elevated cortisol levels and overall depressive symptoms (38), which is consistent with the conclusion of our study.

In addition, this study observed that serum cortisol showed a positive correlation trend with the diurnal variation factor and body weight factor, and a negative correlation trend with the sleep disorder factor, but none of these correlations reached statistical significance. This may be related to the limited sample size and the fact that single-point cortisol detection is insufficient for a comprehensive assessment of HPA function. Previous studies have shown that abnormal cortisol rhythm is involved in the diurnal symptom fluctuations of depression and the regulation of appetite and body weight. The association between cortisol and sleep symptoms is heterogeneous, which can present as a positive or negative trend. Some studies have also reported no statistically significant association (54–56).

This study also has limitations. It is a cross-sectional study without dynamic observation, and the sample size is relatively small. The changes and correlation analysis of the upstream hormones of serum cortisol, namely corticotropin-releasing hormone (CRH) and adrenocorticotropic hormone (ACTH), were not monitored, and its diurnal variation was not detected, which can only reflect the characteristics of samples collected at 8 a.m. Additionally, smoking status, BMI, menstrual-cycle phase and wake time were not taken into account, limiting the generalizability of the research results. There are few assessment tools using both self-administered and rater-administered scales. Conducting research to further improve these conditions is our future direction.

## 6 Conclusions

In drug - naive first - episode patients with major depressive disorder (MDD), a significant increase in serum cortisol levels can serve as one of the biological indicators for correlating with the core symptom clusters and disease severity of MDD. Meanwhile, in the absence of significant breakthroughs in the mechanism of action of existing antidepressants, in the cortisol pathway of the hypothalamic-pituitary-adrenal axis, the regulation of serum cortisol levels may have potential value in ameliorating the symptoms of MDD.

## Data Availability

The original contributions presented in the study are included in the article/**Supplementary Material**. Further inquiries can be directed to the corresponding author.

## Supplementary material

Supplementary Table 1 Diagnostics of the quadratic polynomial regression model for SDS score.
