# Supplementary material for: Correlation between hypothalamic-pituitary-adrenal function and clinical characteristics in the acute phase of first-episode drug-naive major depressive disorder
Source: Front Psychiatry. 2026 Oct 26;17:1985217. doi: 10.3389/fpsyt.2026.1985217 (PMC13746195; doi:10.3389/fpsyt.2026.1985217)
Supplement: Supplementary file 1 [file Table1.docx]

**Supplemental Table**

Table1 Diagnostics of the quadratic polynomial regression model for SDS score

| Category | Indicator | Value |
| --- | --- | --- |
| Model overall fit | Adjusted R² | 0.326 |
|  | ΔR² | 0.047 |
|  | F change | 7.064 |
|  | P (quadratic term) | 0.009 |
| Residual independence | Durbin–Watson statistic | 1.658 |
| Multicollinearity | VIF (cortisol_c) | 1.672 |
|  | VIF (cortisol_c2) | 1.612 |
|  | Maximum condition index | 12.907 |
| Basic residual statistics | Min / max raw residual | −15.4551 / 21.1757 |
|  | Min / max standardized residual | −2.117 / 2.901 |
| Influence statistics | Studentized residual | −2.170 / 3.032 |
|  | Centered leverage | 0.011 / 0.264 |
|  | Cook’s distance | 0.000 / 0.142 |
| Regression coefficients | B (cortisol_c) | 0.038 |
|  | B (cortisol_c2) | 0.000* |
| Turning point | Estimated turning point (centered cortisol_c) | −133.0 |
|  | 95% CI (centered cortisol_c) | −297.90 to 31.90 |
|  | Estimated turning point (raw serum cortisol) | 208.07 nmol/L |
|  | 95% CI (raw serum cortisol) | 43.17 to 372.97 nmol/L |

Note. SDS, Self-rating Depression Scale; VIF, variance inflation factor; CI, confidence interval. The quadratic coefficient displayed as 0.000 results from decimal rounding in the software output; the quadratic term is statistically significant (P = 0.009). Studentized residual, centered leverage, and Cook’s distance are reported as minimum / maximum values across observations. The estimated turning point of 208.07 nmol/L for raw serum cortisol lies within the observed range of raw cortisol values in this sample (n = 103). The 95% CI of the turning point is relatively wide, reflecting uncertainty in the precise location of the minimum point of the quadratic curve. Shapiro–Wilk test for residual normality and Breusch–Pagan test for homoscedasticity are to be reported once computed.
